# Photocurrent response loss of dye sensitized solar cells owing to top surface *nanograss* growth and bundling of anodic TiO_2_ nanotubes

**DOI:** 10.1016/j.heliyon.2024.e24247

**Published:** 2024-01-06

**Authors:** Maryam Yavarzadeh, Farzad Nasirpouri, Leila Jafari Foruzin, Amin Pourandarjani

**Affiliations:** Faculty of Materials Engineering, Sahand University of Technology, Tabriz 51335-1996, Iran

**Keywords:** Backside illuminated dye-sensitized solar cell, TiO_2_ nanotubes arrays, Single step anodization

## Abstract

In this research, the effect of anodization time on the length of the titanium oxide nanotube arrays (TNAs) and photovoltaic parameters of back-side illuminated dye-sensitized solar cells (DSSCs) were investigated. The TNAs were characterized using X-ray diffraction (X-ray) or (XRD), and scanning electron microscopy (SEM). Anodic TNAs having tube lengths from 7.9 to 20.17 μm were produced in ethylene glycol containing ammonium fluoride-NH_4_F by increasing the anodizing time from 20 min to 6 h. Based on I–V curves, the power conversion efficiency (PCE) of back-side illuminated dye sensitized solar cells (DSSCs) increased for TNAs grown for up to 120 min, but decreased afterward. Using electrochemical impedance spectroscopy (EIS), we understand that the resistance of the TNAs decreased from 94.82 Ω cm^−2^ for TNAs anodized for 20 min down to 50.43 Ω cm^−2^ for those TNAs anodized for 120 min, however, it increases for TNAs anodized for longer periods of time. Furthermore, the short circuit current density (J_sc_) increased from 3.14 to 5.67 mA cm^−2^ during 2 h anodic oxidation for TNAs, and leading to enhanced efficiency of about 200 % (from 1.19 % to 2.45 %). We interpret this behaviour with the top surface morphology evolution of TNAs as a function of anodization time which is associated with the formation of top surface nanograss and bundling the tubes for specific durations.

## Highlights

• The nanograss growth was controlled by anodization in solution contain NH_4_F and ethylen glycol.
• Optimized titanium nanotube arrays (TNAs) exhibited excellent efficiency at back-side illuminated DSSC cell.
• During optimized time for anodization (2h) the short circuit current density (J_sc_) increased from 3.14 to 5.67 mA cm^−2^.
• At TNAs, enhanced efficiency of about 200 % (from 1.19 % to 2.45 %).

## Graphical abstract

**Figure:**
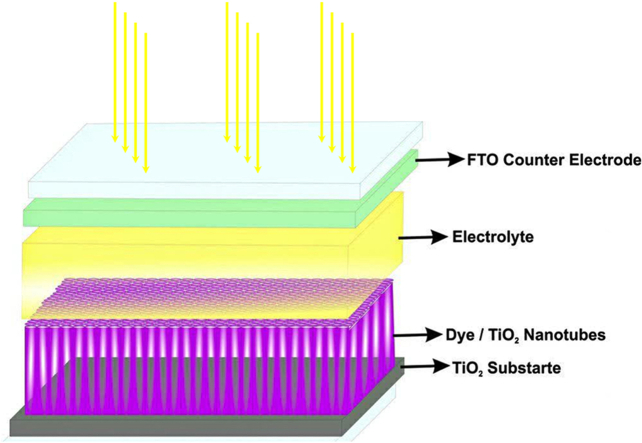

## 1 Introduction

Nanoscale materials are among the most interesting novel materials due to their exceptional physicochemical properties and large surface area [1,2]. Among various nanostructured materials, TiO_2_ was an interesting material for functional and device applications owing to its unique electronic properties [3], very high specific surface area [4], high mechanical strength [5], and high electron mobility [6,7]. Among all the metal oxides, TiO_2_ nanotubes (TNAs) as a wide-band gap nanostructured semiconductor is the most extensively studied candidate for optoelectronic and solar cell devices. TNAs can be fabricated by a simple electrochemical method namely anodic oxidation [8].

Dye sensitized solar cells were first introduced in 1991 by O'Regan Gratzel, where TiO_2_ was used as a photoanode [9]. TNAs have a tunable band gap energy and suitable band-edge position that makes them a very important material as anode based materials in dye sensitized solar cells (DSSC) [10,11]. The efficiency of DSSCs depends in some aspects on the electron transport properties in the photoanodes [12,13]. One dimensional TNAs are known as the material of choice for this purpose with long electron pathways and rapid electron transport. The geometry, thickness, outer and inner diameters and the length of nanotubes can be controlled during anodic oxidation by adjusting the time, voltage, and electrolyte content [8,14].

High surface area considerably manipulates the efficiency of DSSC [15]. Yi and co-workers, reported that the increase in the thickness of TNAs enhances the surface area [16]. Anodization voltage and time are two important factors that affect the thickness of TNAs [8,17]. Xie and co-workers reported that the voltage of anodization method mostly controls the pore diameter of nanotubes while it showed low relationship with the length of TNAs [14]. Longer nanotubes lead to the high surface area but increase the pore diameter and decrease the number of TNAs. With increasing the anodization time, the length of TiO_2_ nanotube arrays increases [18]. The nanotube length fabricated by the anodic oxidation method can be ranged from a few hundred nanometers up to 60 μm [19]. Some studies illustrated that with increasing the anodization time, the photoactivity is increased [20].

Recombination rate of electrons is another critical parameter for controlling the performance of DSSCs. In order to increase the power conversion efficiency (PCE) of DSSCs, the dye regeneration occurs much faster than the recombination of electrons with the oxidized dye molecules. Therefore, the recombination reaction is as an inefficiency source, because it leads to the internal short-circuiting of cell [21,22]. The time of charge transport through the TNAs may also influence the nanostructure of TNAs. In addition, TNAs absorbing more dyes act as a transport media for the injected electrons from dye molecules. One of the fastest chemical processes in DSSC is electron injection between conduction band of TiO_2_ and dye molecules’ excited band. The rate of recombination of charge between conductive band and injected electron should be slower than rate of electron injection. Thus, the electron regeneration should occur in rapidly enough compared with the recombination of electrons [23]. Different methods have been performed to reduce the recombination rates one of which is the application of TNAs with longer lengths [24,25]. With increasing the length of nanotubes, the efficiency of DSSCs was shown to be improved. Long TNAs absorb more amount of loading dyes and consequently can absorb more incident photons [19]. Recent research reported that the length of TNAs and the efficiency of DSSC increased linearly with the anodizing time and the efficiency of DSSC at 7h anodization reached to the 1.96 % [16]. However, Lou et al. [26] reported the efficiency of DSSC increases with enhancement of nanotubes (in the range of 14.3–19.2 μm) until reaching a maximum value at the 18.3 μm and then shows a decrease at efficiency. However, TNAs used in DSSC must be sufficiently long length to adsorb large amount of dyes but with increase the time of anodization, some bundling (nanograss) and micro-cracks are formed at long TNAs and decrease the efficiency of DSSC.

**Fig. 1:**
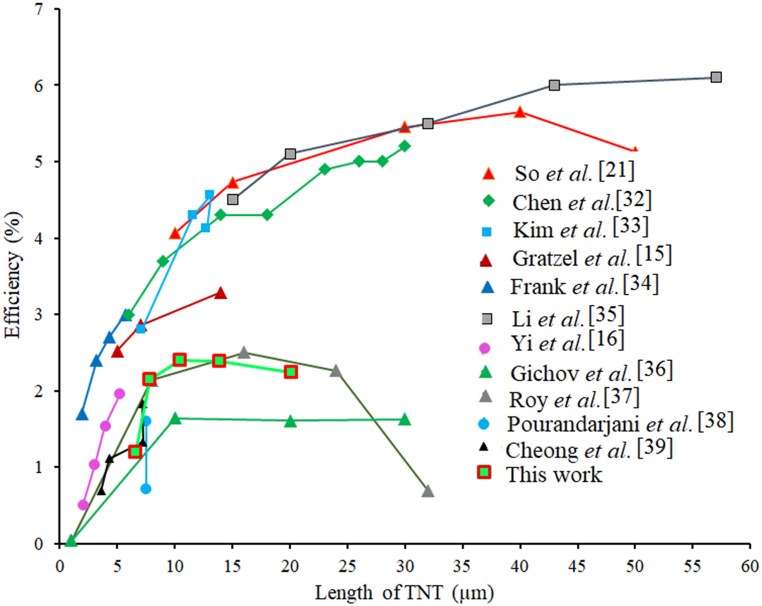
The trend of solar cell PCE values for TiO_2_ nanotube arrays (TNAs) based backside illuminated DSSCs as a function of nanotube length extracted from earlier reports and compared with teh PCE enhancement achieved in the present work.

Our previous reports [27] mentioned that the TNA photoelectrodes prepared by two-step anodization process (first step: 6 h and second step: 1 h) on electropolished Ti foils, exhibited an enhanced photovoltaic performance, compared to those prepared by one step anodization, owning to the enhanced electron lifetime and light harvesting. The maximum efficiency of backside illumination DSSCs for the two-step anodization process (first step: 6 h and second step: 1 h) was about 0.73 %. In 2006, Paulose and co-workers, reached the efficiency of backside illumination DSSCs about 4.24 %. They used one step anodization for 7 h and the length of TNAs was reported about 6 μm [28]. Momeni and co-workers, used two types of electrolytes to synthesize TiO_2_ nanotube arrays by one step anodization. The efficiency of TNAs anodized by one step anodization in the aqueous electrolyte reached 0.63 %. The anodizing time was about 2h nevertheless the PCE for TNAs anodized in organic electrolyte for 8h reached 1.176 % [29]. In 2012, Nair and co-workers, reported that after anodizing for 24 h the length of nanotubes was increased to 1.5 μm. In addition, they used Titanium tetrachloride-TiCl_4_ treatment for photoanodes used in backside illumination DSSCs and reached the maximum efficiency about 3.5 % for photoanodes which anodized during 24 h [30]. Choi and co-workers, reported that TNAs with length about 15 μm after treatment Titanium tetrachloride-TiCl_4_ can be obtained the efficiency about 2 % at backside illumination DSSCs. Last but not the least, the efficiency of front side illumination DSSCs is generally higher than that of backside illumination DSSCs [31]. Still the improvement of efficiency at backside illumination DSSCs is an important challenge. Therefore, decreasing the time of anodization is a key factor for increasing the efficiency of TiO_2_ nanotube based DSSCs. There has been a great motivation to enhance the features of the cell's components to improve the efficiency to even more magnitudes. The trend of the efficiency values for backside illuminated DSSCs with increasing the length of TNAs from earlier works is represented in Fig. 1 [15,16,21,[32], [33], [34], [35], [36], [37], [38], [39]].

Our new work is the resumption of our previous work with better conversion efficiency. In the present work, a systematic study was performed to exploit the effect of growth regularities and irregularities happening during the anodization process for different times. We prepared TiO_2_ nanotube arrays by one step anodization and optimized the time of anodization in order to achieve high efficiency in backside illuminated DSSCs. A series of TiO_2_ nanotubes were prepared under different anodization times from 20 to 360 min. The effect of length of TiO_2_ nanotubes on efficiency of backside illumination dye sensitized solar cell was studied.

## 2 Experimental

### 2.1 Preparation of TNA films

One-step anodic oxidation was used to obtain TNA s from Ti films. In this study, titanium sheets (Grade 2, 99.7 %, 1 mm thick) were used in all of experiments. The Ti sheets into 1 × 2 cm^2^ films were cut and then put in etchant solution (HNO_3_:HF with volume rater 3:1) for 3 s, then washed out with water and ethanol 99 % respectively. The prepared films then were utilized as the anode in the anodization. The solution containing ethylene glycol (C_2_H_6_O_2_), 0.15 M NH_4_F and 3 vol% deionized (DI) water was used as the anodizing electrolyte. The anodization process was carried out in a two-electrode system (Ti and stainless steel as the anode and the cathode respectively). The samples were anodized at a voltage of 60 V and temperature of about 30 °C in one step for different times including 20, 60, 120, 240, and 360 min whose samples were labeled as N20, N60, N120, N240, and N360, respectively. During anodization process, magnetic stirrer was applied for the stir of solution. The as-prepared samples were annealed at 480 °C. The obtained TNAs were investigated by the backside illuminated *DSSC*.

### 2.2 Preparation of DSSCs

TNA films were used as the photoanode and platinum coated florin coated tin oxide (FTO) sheets were used as the cathode and as the light entrance as well. TNAs were dried at a temperature of 150 °C for 1h. Next, the TNAs were calcined at a temperature about 450 °C for 45 min. The prepared samples were placed in a standard dye solution (N719; 0.4 mM) for 18 h. The spacer was placed between of platinum coated FTO and dye adsorbed TNAs which were subsequently clamped into an integarted unit cell. The iodide electrolyte was injected in the interlayer spacer of the cell using vacuum system and hole of cathode by transparent paste was closed. The same conditions were used for fabrication of all samples and there are no other parameters interfered.

### 2.3 Physical characterization

During anodization, a digital multimeter connected to computer *via* an A/D data acquisition system was used to measure the current changes. Scanning electron microscopy (SEM) model Mira 3-XMU was employed to examine the morphology of TNAs. The inner diameter and length of TNAs were measured using *Image J software*. X-ray diffraction (XRD) or (X-ray) patterns were investigated using Brucker equipment model D8-Advance, using Cu kα radiation (voltage = 40 kV, current = 35 mA and wavelength = 0.154178 nm). For recording of the photovoltaic parameters of such as J_sv_ and V_oc_, a solar spectrum simulator under illumination 1.5 Am with a power of 100 W/cm^2^ was used. Also, a computer-controlled potentiostat (model OrigaFlex-OGF500) was used for electrochemical tests. The electrochemical impedance spectroscopy (EIS) analysis of obtained DSSCs was investigated using OrigaFlex-OGF500 at frequencies ranging 1–100 Hz.

## 3 Results and discussion

### 3.1 Anodization method for preparation of TNAs

**Fig. 2:**
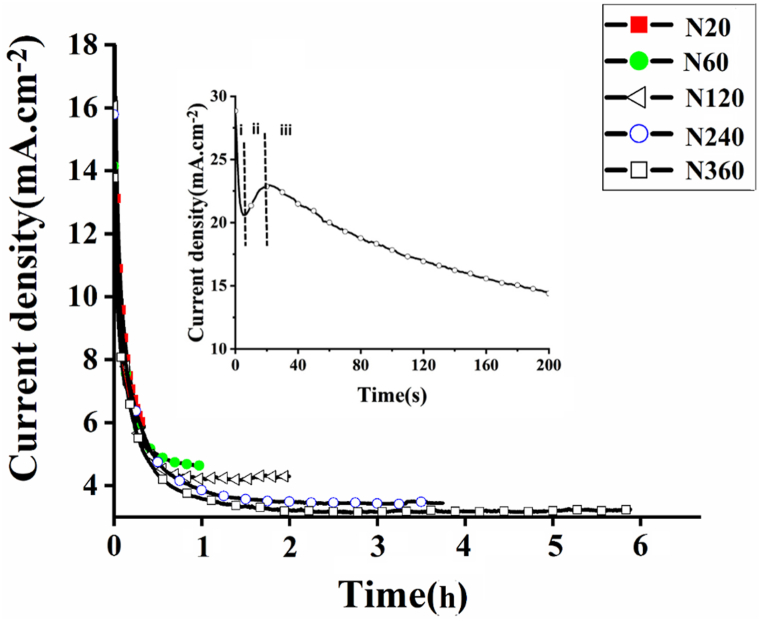
First section of the current density transient curve of anodic oxidation for 1h. The inset shows a typical current transient highlighting three steps of TNA formation separated qualitatively by dashed lines.

Fig. 2 shows initial section of the current transient (J-t) measured during single-step anodization *process* under different anodization times of 20, 60, 120, 240 and 360 min. As mentioned in the literature [[40], [41], [42]], three stages typically exist for the growth of TNAs. During stage *I*, a current density drop is observed due to the formation of a thin barrier oxide dielectric layer. Furthermore, fluoride ions cause the formation of pits in the oxide film grown initially. Anodic current density starts to increase due to formation of the pits in the barrier layer as observed in stage *II*. These pits become longer and deeper and consequently the pores are formed that will extend through the surface by prolonging the process. At end of the pores where the electric field is present the oxide layer becomes thinner, so-called field-assisted dissolution (FAD) which affects the dissolution of oxide layer in preferential sites, e.g. pits. FAD mechanism interprets the dissolution of the oxide layer at end of the pore and diffuse to the Ti substrate. The chemical dissolution and oxidation can be active at the bottom end of the pores. Top of the formed pores starts to be removed by chemical dissolution and on the other hand the surrounded oxides grow by the effect of plastic flow. Eventually, as the oxidation and dissolution and oxidation happen simultaneously the nanotubes grow too. As the nanotubes grow longer, a decrease in current density can be seen [38,43]. Table 1 reports the current density applied during time of anodic oxidation for 20, 60, 120, 240, and 360 min and the thickness or the length of the tubes grown.

**Table 1:** Anodic oxidation duration and the current density of TiO_2_ arrays recorded during anodization at different times.

Samples	Anodic oxidation time(min)	Current density(mA.cm^−2^)	Length of TNAs (μm)		Error (μm)
N20	20	5.87	7.90		±0.07
N60	60	4.63	8.50		±0.07
N120	120	4.29	10.43		±0.10
N240	240	3.44	13.91		±0.15
N360	360	3.24	20.17		±0.20

It has been demonstrated that nanograss formation on top of the tubes which blocks the ion transport channels limits the mass transport entirely. Therefore, the anodization process, happens under a limited ionic mass transport and the kinetics of dissolution and formation of TNAs slows down [40]. After a long time of growth, the irregularity takes place in the tube wall which leads ultimately to rough topography on the surface. In this step, small oxide patches formed on top of TNAs seem to block the tube opening [16]. Fig. 2 shows that the formation of nanotubes with longer length with irregular and disordered structure on top of the nanotubes. This is consistent with the reduced current density recorded during anodization process as the anodization time increases. As shown in Table 1, the current density of anodization was decreased, if the time of anodization increased from 20 to 360 min, as is evident from highest current density of 5.87 down to 3.23 mA cm^−2^.

### 3.2 Morphology of TNAs

**Fig. 3:**
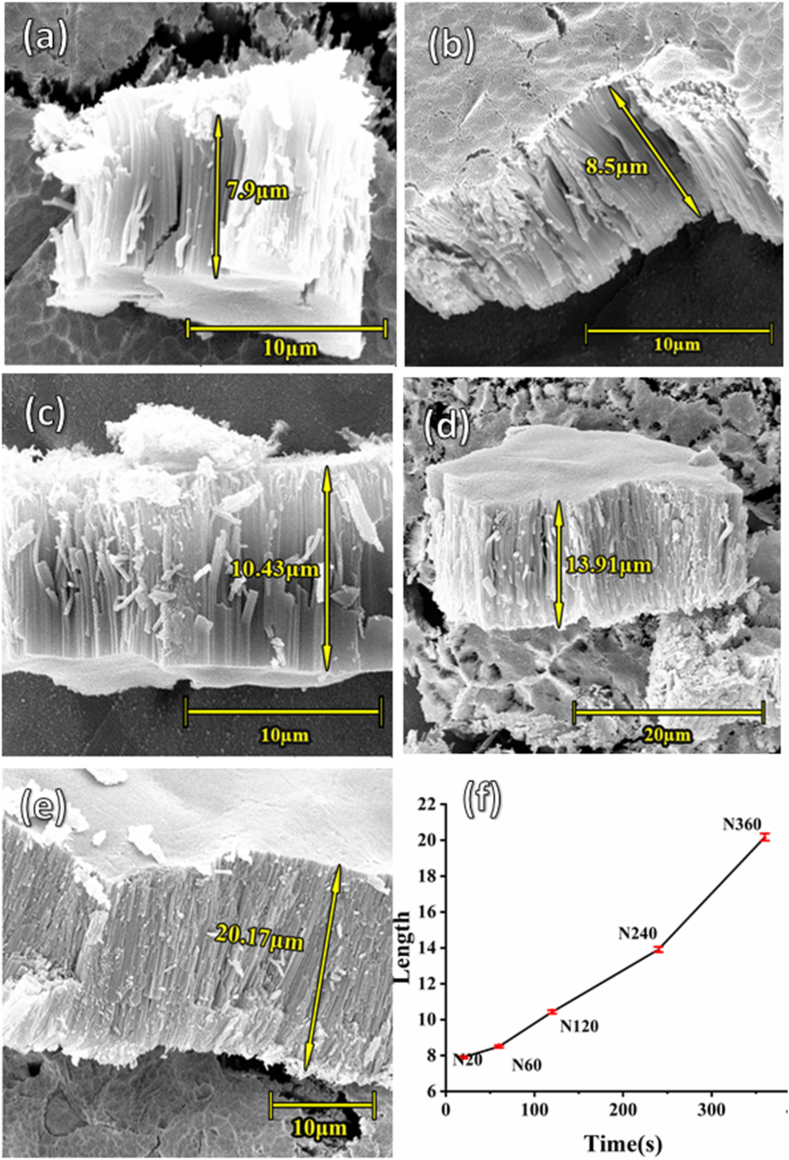
Cross-sectional SEM images of TNAs grown with different anodic oxidation time (a) N20, (b)N60, (c)N120, (d)N240 and (e)N360 and (f) the time-length curve for anodized samples. HV: 20 kV, SEM MAG of (a, b, c, e, and f): 10 kX and SEM MAG of (d): 5 kX.

The cross-sectional SEM images of nanotubes with different anodization times are shown in Fig. 3. The length of the nanotubes anodized for different durations (20, 60, 120, 240 and 360 min) is 7.9, 8.5, 10.43, 13.91, and 20.17 μm, respectively as reported at Table 1. The data were collected from precise measurements on SEM images as depicted in Fig. 3(a), (b), (c), (d), and **(e)**. It was reported that the length of TNAs depends on an interplay between two factors of electrochemical etching and chemical dissolution of TNAs. With increasing the anodization time the etching rate increases too much until reaches a balanced state [26]. As shown in Fig. 3(f), there is a linear relationship between increasing the length of the TNAs and increasing the anodizing time. Fig. 3(f) shows the variation of TNA length as a function of anodization time, indicating that with increasing the anodization time about 18 times (from 20 to 360 min) the length of TNAs increases about 2.5 times (from 7.9 to 20.17 μm).

**Fig. 4:**
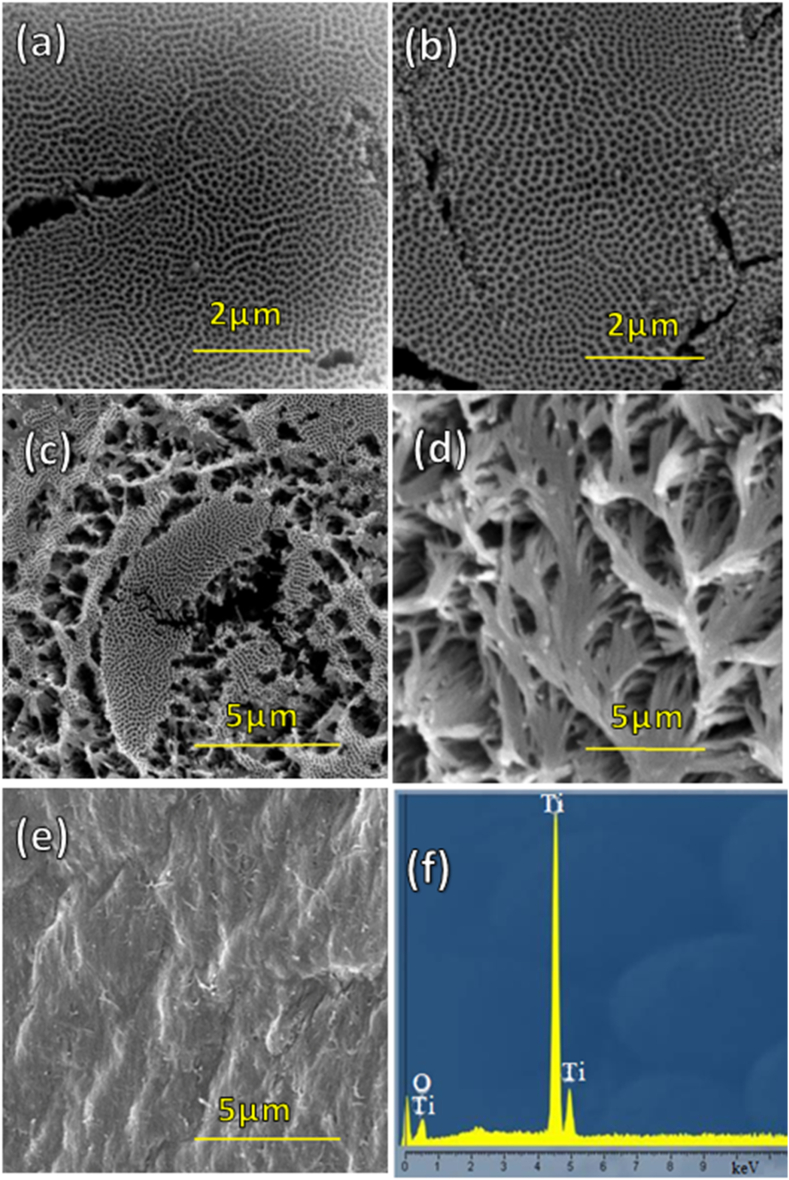
SEM images taken from top of TNAs anodized for different anodization times (a) 20min (b) 60 min (c) 120 min (d) 240 min (e) 360 min and (f) EDAX analysis of TNA (HV: 20 kV, SEM MAG of (a, b, and c): 30 kX, and SEM MAG of (d, e, and f): 15 kX).

Fig. 4 shows SEM images taken from top region of TNAs grown. As is evident from Fig. 4(a), and (b), the formation of nanograss not seen in shorter TNAs anodized only for 20 and 60 min and consequently, TNAs have an ordered structure. In contrast, as shown at Fig. 4(c), when the length of TNAs exceeds 10.43 μm, the TNAs form close-packed clusters at top region leading to bundling of the tubes and an irregularity known as nanograss. With increasing the time of anodizing to 240 and 360 min, the growth of nanograss was more pronounced, Fig. 4(d) and (e). The main reason for formation of nanograss is due to over etching of TNAs top parts. Lee et al. reported that the chemical etching leads to thinning of the tube wall at top followed by falling of tubes resulting in some grass-like structure at the top [44]. It is particularly important in dye sensitized solar cells that the formation of nanograsses on top of the tubes blocks the dye penetration and absorption and are unfavorable for efficient light absorption and electron transport when used in photovoltaic devices as will be discussed in later section. Furthermore, the EDAX analysis of TNAs is reported in Fig. 4(f). As reported, the presence of titanium and oxygen confirmed the preparation of TNAs without any impurity.

### 3.3 Crystal structure

**Fig. 5:**
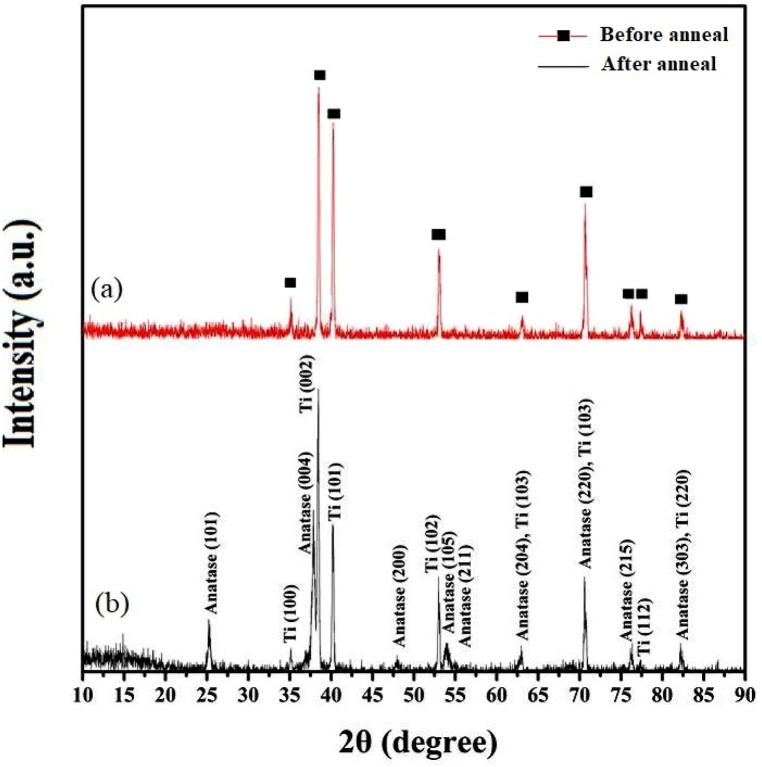
X-ray diffraction patterns of TiO_2_ TNAs before and after annealing at 450 °C.

An important factor that affects the electronic band gap of TNAs is the crystal structure of titanium oxide. In order to investigate the crystal structure of anodized TNAs in NH_4_F electrolyte, the samples have been annealed in 450 °C. X-ray diffraction pattern of TNAs before and after annealing is presented in Fig. 5(a), and **(b).** As shown in Fig. 5(a), anodized TNAs before annealing have amorphous structure and they only have titanium diffraction peaks. There is no crystalline phase observed in as-anodized TNAs indicating of their amorphous structure. This correlated to standard card (JCPDS No. 44–1294) [45]. With annealing in the range of 450 °C as the same condition for all samples the anatase crystalline phase was appeared, and the Bragg peaks related to anatase can also be seen in Fig. 5(b). These peaks include (101), (004), (200), (105), (211), (204), (215), (303) and matched with standard card (JCPDS No. 21–1272) [46].

### 3.4 Backside-illuminated DSSCs performance

**Fig. 6:**
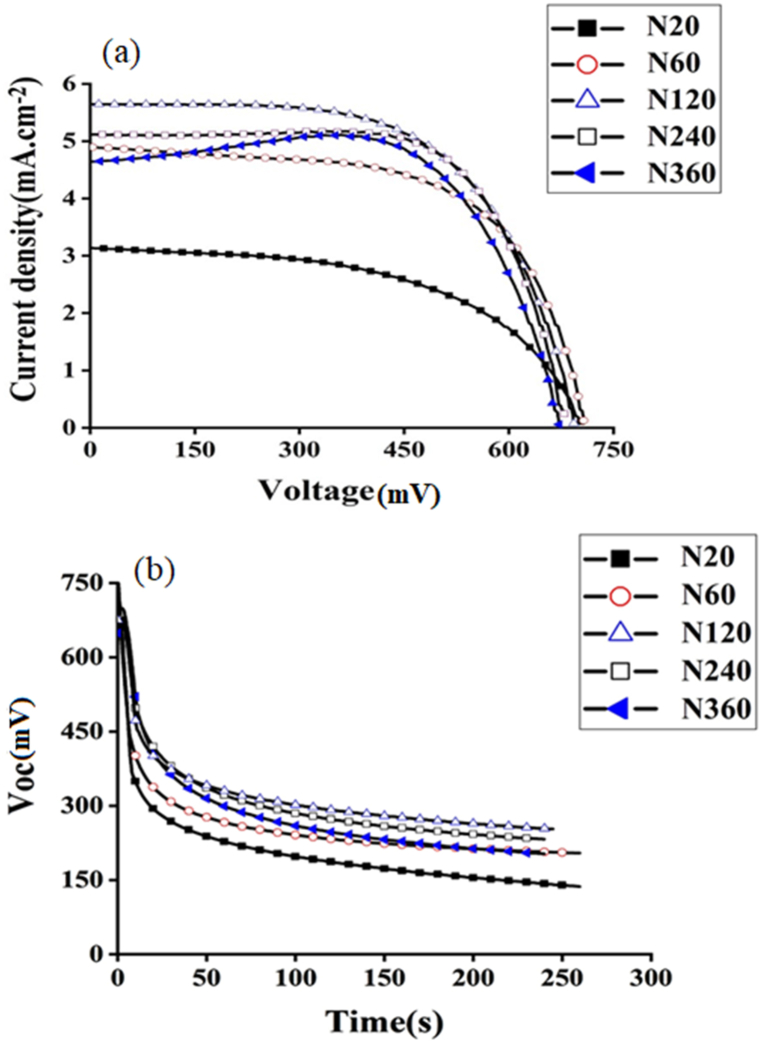
(a) Current density-voltage (I-V) curves and (b) open circuit voltage- time (V_oc_ -t) curves of DSSCs prepared using TNAs with different andization times or lengths.

Fig. 6(a) and **(b)** show photocurrent density-voltage (J-V) and V_oc_ decay curves. As shown at Fig. 6(a), the photocurrent density at TNA-N60 is higher than that other. The photocurrent density at TNA-N20 is lower than that others. At TNA-N360 in the voltage region of 200–450 mV, a small hump was reported. This hump was originated from recombination current which decreased the efficiency of TNA-N360 [47,48].

**Fig. 7:**
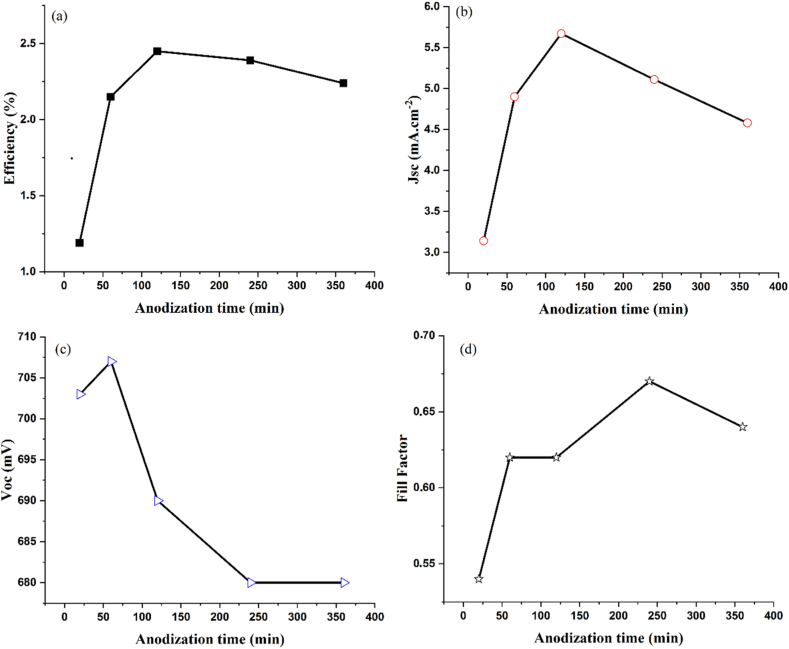
Photovoltaic data (a) PCE, (b) J_sc_, (c) V_oc_, and (d) fill factor to comapre the DSSC performance prepared using TNAs with different andization times or lengths.(lines are a guide to eyes).

The short-circuit current density (J_sc_) and open circuit voltage (V_oc_) were obtained from the J-V curve **[(**Fig. 6(a)**]**, and the fill factor (FF) and power conversion efficiency (PCE) were calculated using the value of J_sc_ and V_oc_. The data extracted from the plots are summarized in Table 2 and for better comparison replotted in Fig. 7(a–d). As shown, all the parameters of DSSCs depend significantly on the time of anodization time or the length of TNAs. The conversion efficiency and short circuit current density both increase with increasing of the nanotube length at the beginning and then decrease subsequently. This is due to the fact that TNAs growth is accompanied with morphology change, where the tubes exhibit regular geometry and structure at the beginning whereas irregularities are introduced for longer nanotubes. TNA arrays with regular and longer tube lengths certainly offer more surface area for loading dye molecules which allows better harvesting of light, yielding higher current density of DSSC [49]. We find that a critical length of TNAs exists here for those anodized for 2 h with a nanotube length of 10.43 μm where a change of DSSC performance was observed. It is interestingly consistent with the diffusion length of electrons in this TiO_2_ layer >10 μm reported in the literature [50]. This sample provides the best photoelectron diffusion length where the efficiency of DSSC is maximum whose PCE reaches 2.4 %. Our results could be interpreted by considering either the tube geometry or the progression of recombination of electrons. Although the tube irregularities are introduced due to the formation of nanograss after anodizing for more than 2 h, it must be noted that once the length of TiO_2_ nanotube exceeds the photoelectron diffusion length, the electron recombination in the interface of electrode and I^−^/I^−3^ redox occurs, so the collection of photogenerated carries becomes low, and consequently, leads to the poor performance of DSSC [51]. Therefore, electrons encounter more trapping and de-trapping in longer pathways, which causes to decrease the DSSC efficiency [27]. With increasing the anodization time, short circuit current density increases to the 5.67 mA cm^−2^ at 120 min anodization time and then decreases for longer periods of anodization. This can be attributed to this phenomenon that longer nanotubes have large surface area and adsorb more dyes and therefore, a higher number of photogenerated electrons exist, but according to Fig. 3, when the length of nanotubes is longer than 10.43 μm (after 120 min anodization time), some irregular porous oxide layer or nanograss covers the top of TNAs, which remarkably influences the electron transport and consequently, decrease the J_sc_. In one dimensional photoelectrode, electrons move along the length of TNAs but when nanograsses form at top of the tubes it causes intertube contacts. Therefore, electrons may move in transverse or longitudinal directions. Thus, the transport pathway becomes longer and electrons fall in more traps. Longer electrons pathway causes more trap states and more recombination. The variation of fill factor as a function of TNA length seems not affect the DSSC performance here. The fill factor is determined by interface junction between the surface of TNAs/Dye and the electrolyte which may impose large nonlinear resistance in the cell. If not high enough, the holes resulting from the exciton dissociation at the semiconductor/liquid junction are not dispatched from the surface at a highly enough rate. Other determining parameters influencing fill factor ate the energy level of the ions and the their concentration which may introduce series resistance in the cell. The increasing trend of the fill factor in our cells could be interpreted by loading more dye molecules on longer and rougher TNAs and consequently better light harvesting and import no negative effect on the cell performance as a function of TNA length discussed here. Another parameter is the energy difference between the TiO_2_ level of energy (Fermi energy) and the electrolyte level of energy (Nernest energy) defined as the open circuit voltage (V_oc_). V_oc_ for all fabricated cells is approximately the same in meaningful range of 0.69 ± 0.2 V. As evident from Table 2, with increasing the anodization time, V_oc_ firstly increases and then decreases. The reduction of V_oc_ demonstrates decrease of electron density in longer nanotubes which may be caused by the decrease of light absorption on longer nanotubes. A possible reason for decreasing of V_oc_ is increasing the intermolecular π-π interaction between neighboring dye molecules in long TiO_2_ nanotubes that decreased the value of V_oc._ [52]. Open circuit voltage decay curve for all cells are presented in Fig. 6(b). V_oc_ for cells that anodized to time of 120 min, open circuit voltage decay is slower than that other photoanodes. This means these cells possess higher electron lifetime and lower recombination rate. The electron lifetime can be obtained by the equation below [53]:

(1) $$\tau_{n}=\left(\frac{{-TK}_{B}}{e}\right)\;{\left(\frac{dVoc}{dt}\right)}^{-1}$$

where K_B_ is the Boltzmann constant, T is the temperature, e is the positive elementary charge.

**Table 2:** -Photovoltaic data of fabricated DSSCs using TNAs.

Sample	Anodization time (min)	Length of TNA (μm)	J_sc_ (mA.cm^−2^)	V_oc_ (mV)	Efficiency (%)	Fill Factor
N20	20	7.90	3.14	703	1.19	0.54
N60	60	8.50	4.90	707	2.15	0.62
N120	120	10.43	5.67	690	2.45	0.62
N240	240	13.91	5.11	680	2.39	0.67
N360	360	20.17	4.58	680	2.24	0.64

**Fig. 8:**
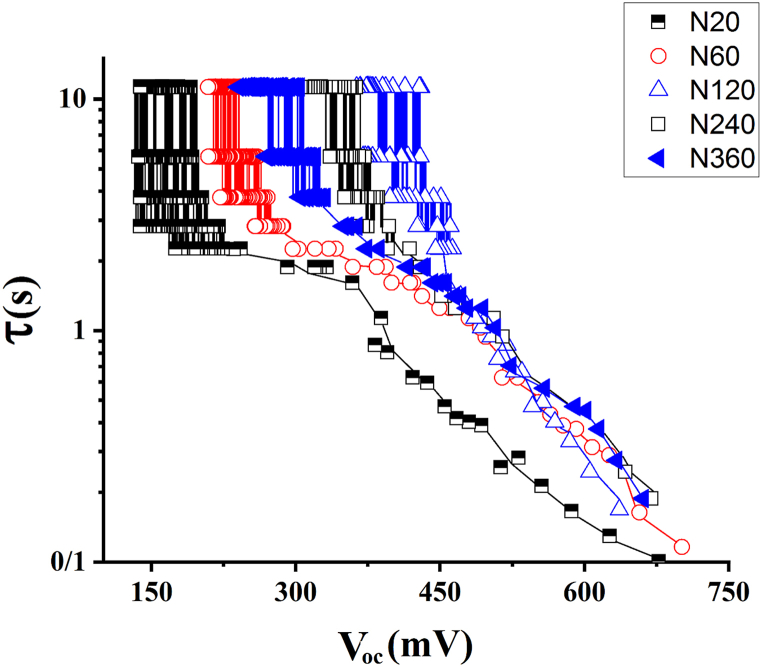
Electron lifetime curves as a function of V_oc_ at TNAs prepared with different anodization times.

The correlation between the calculated $\tau_{n}$ and V_oc_ is shown in Fig. 8. Increasing the length of anodized TNAs, the electron lifetime has increased. Larger electron lifetime means slower recombination rate [27,54]. We observe that the TiO_2_ nanotubes anodized for 2 h have slower recombination rate compared to other TNAs and has the best photovoltaic performance.

**Fig. 9:**
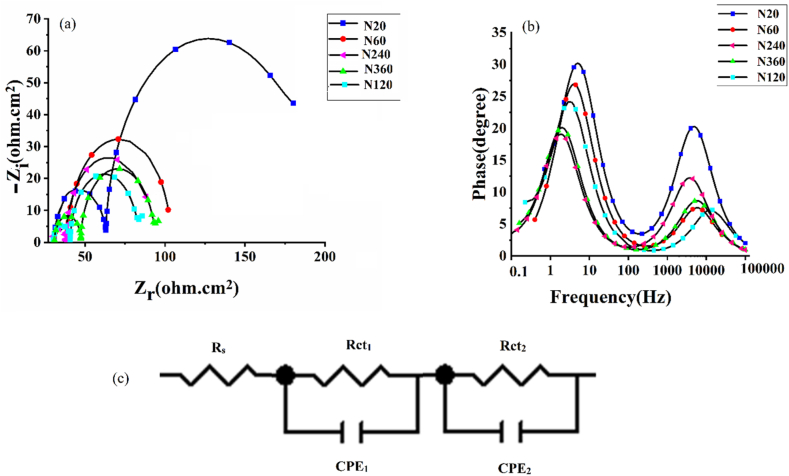
EIS analysis: (a) Nyquist and (b) bode plots of DSSCs fabricated using TNAs prepared with different anodization time, and (c) their equivalent circuit for EIS data.

The performance of DSSC was analyzed by electrochemical impedance spectroscopy (EIS) to study of the charge transport behavior of TNAs in the DSSC. The corresponding results are reported **in** Fig. 9 **and the parameters obtained by fitting the EIS results are presented in** Table 3**. The electron lifetime can be calculated from the maximum frequency of second semi-circle (f**_**max**_**) in Nyquist plot** [49]**. Based on** Fig. 9(a)**,** the values for *f*_max_ were obtained respectively 2.81, 2.51, 1.25, 1.58, 1.77 Hz for anodized photoanoodes for five different times of 20, 60, 120, 240 and 360 min and the electron lifetime was estimated using the following equation [55].

(2) $$\tau =\frac{1}{2\pi \;f_{\max}}$$

where τ is value for electron lifetime, *f*_max_ is maximum frequency of second semi-circle in nyquist plot. The first semicircle corresponds to the capacitance and resistance of the interfacial layer while the second semicircle corresponds to the double layer capacitance and charge transfer resistance. Therefore, electron lifetime must be calculated from the maximum frequency corresponding to the peak of the second semicircle [56,57]. The value of electron lifetime for samples anodized for five different times of 20, 60, 120, 240 and 360 min are measured about 56, 63,127, 100 and 90 ms, respectively. Furthermore, the Niqest curves of prepared samples are reported in Fig. 9(a). Also, the correlated bode plots was reported at Fig. 9(b). As shown the radius of the circle for TNAs anodized for 2 h (sample N120) is smaller than that of other TNAs. Thus, TNAs anodized for 120 min possesses less charge transfer resistance, R_ct_, which is defined as a resistance attributed to charge transport at the interface of TiO_2_/electrolyte/dye in DSSCs [58]. The fitted values for R_ct_ according to Table 3, reveal that with increasing the anodization time up to 120 min, R_ct_ decreases and the recombination slows down. The best coherency of the components TiO_2_, dye molecules and electrolyte, can be the result of lower R_ct_ in N120 cells, in shorter TNAs such as TNA-20 and TNA-60, there is little space for dye molecules and electrolyte to stablish a sufficient interface and as the SEM images (Fig. 3) illustrates for longer TNAs (TNA-240 and TNA-360) the order of the structure collapses and formation of nanograss spoils the coherency and interface of the components. An equivalent electrical circuit is presented in Fig. 9(c). The model of the equivalent circuit consists of constant phase element (CPE_1_) in parallel with R_ct1_ and CPE_2_ which is in parallel with the R_ct2_.

**Table 3:** EIS parameters of DSSCs prepared with TNAs with different anodization times obtained fitting to their electrical equivalent circuit.

Samples	R_s_ (Ω.cm^2^)	R_ct1_ (Ω.cm^2^)	R_ct2_ (Ω.cm^2^)
N20	25.2	35.0	120.0
N60	25.0	15.0	65.1
N120	30.0	10.0	42.0
N240	28.0	12.0	47.0
N360	30.0	17.0	46.5

## 4 Conclusion

We concluded that in one step anodic oxidation method of titanium oxide nanotubes (TNAs) for different times, the length of TNAs exhibit remarkable impact on the photo to current conversion efficiency in DSSCs. The power conversion efficiency (PCE) of TiO_2_ nanotubes as the photoanode in back side illuminated DSSC reaches the maximum efficiency of 2.40 % during 120 min of anodization where the length of TNAs is 10.43 μm. This is aworth noting that as a consequence of increasing the anodization time more surface area will be available for loading more dye molecules. However, further increase in the anodization time from 120 to 360 min changes the length of nanotubes from 10.43 to 20.17 μm. The top surface of TNAs start to be bundled which form nanograsses on the top surfaces. Thus, the PCE starts to decrease as the anodic time increases up to 360 min. As the anodization time increases up to 120 min the recombination rate of electron-holes slows down and there is more chance to prolong the electron lifetime. Therefore, as a result, the anodization time is a critical parameter for the production of TNAs as the photoanode in DSSCs exploited here in the backside illumination dye sensitized solar cells.

## CRediT authorship contribution statement

**Maryam Yavarzadeh:** Methodology, Investigation, Validation, Visualization, Writing – original draft. **Farzad Nasirpouri:** Conceptualization, Data Curation, Resources, Writing – review & editing, Project administration, Supervision, Funding acquisition. **Leila Jafari Foruzin:** Conceptualization, Methodology, Writing – original draft, Writing – review & editing, Investigation, Validation, Visualization. **Amin Pourandarjani:** Investigation, Validation, Visualization, Writing – review & editing.

## Declaration of competing interest

The authors declare that they have no known competing financial interests or personal relationships that could have appeared to influence the work reported in this paper.
